# Crosstalk of Nrf2 with the Trace Elements Selenium, Iron, Zinc, and Copper

**DOI:** 10.3390/nu11092112

**Published:** 2019-09-05

**Authors:** Maria Schwarz, Kristina Lossow, Johannes F. Kopp, Tanja Schwerdtle, Anna P. Kipp

**Affiliations:** 1 Department of Molecular Nutritional Physiology, Institute of Nutritional Sciences, Friedrich Schiller University Jena, Dornburger Str. 24, 07743 Jena, Germany; 2 TraceAge-DFG Research Unit on Interactions of Essential Trace Elements in Healthy and Diseased Elderly, D-13353 Potsdam-Berlin-Jena, Germany; 3 German Institute of Human Nutrition, Arthur-Scheunert-Allee 114-116, 14558 Nuthetal, Germany; 4 Department of Food Chemistry, Institute of Nutritional Science, University of Potsdam, Arthur-Scheunert-Allee 114-116, 14558 Nuthetal, Germany

**Keywords:** Nrf2, selenium, iron, copper, zinc, homeostasis

## Abstract

Trace elements, like Cu, Zn, Fe, or Se, are important for the proper functioning of antioxidant enzymes. However, in excessive amounts, they can also act as pro-oxidants. Accordingly, trace elements influence redox-modulated signaling pathways, such as the Nrf2 pathway. Vice versa, Nrf2 target genes belong to the group of transport and metal binding proteins. In order to investigate whether Nrf2 directly regulates the systemic trace element status, we used mice to study the effect of a constitutive, whole-body Nrf2 knockout on the systemic status of Cu, Zn, Fe, and Se. As the loss of selenoproteins under Se-deprived conditions has been described to further enhance Nrf2 activity, we additionally analyzed the combination of Nrf2 knockout with feeding diets that provide either suboptimal, adequate, or supplemented amounts of Se. Experiments revealed that the Nrf2 knockout partially affected the trace element concentrations of Cu, Zn, Fe, or Se in the intestine, liver, and/or plasma. However, aside from Fe, the other three trace elements were only marginally modulated in an Nrf2-dependent manner. Selenium deficiency mainly resulted in increased plasma Zn levels. One putative mediator could be the metal regulatory transcription factor 1, which was up-regulated with an increasing Se supply and downregulated in Se-supplemented Nrf2 knockout mice.

## 1. Introduction

Essential trace elements (TEs) are micronutrients with indispensable roles in enzymatic reactions, which consequently modify signaling pathways. The effects of TEs are mostly attributed to their redox-modulatory properties. TEs, such as Cu, Zn, and Fe but also Se, can act as pro-oxidants if present in excess or if available as free unbound ions. Otherwise, antioxidant and protective enzymes, such as the selenoproteins, glutathione peroxidases (GPX) and thioredoxin reductases (TXNRD) and also Cu/Zn superoxide dismutase (SOD1), catalase, and metallothioneins (MT), depend on the supply with specific TEs. Via both ways, TEs have the potential to influence redox-modulated signaling pathways. Until now, many transcription factors have been shown to be sensitive towards the cellular redox status. Among them, nuclear factor erythroid 2 p45-related factor 2 (Nrf2) is a better characterized one [1].

Under basal conditions the transcription factor Nrf2 is kept in the cytosol by its binding partner, Kelch-like ECH-associated protein 1 (Keap1), which is anchored to the actin cytoskeleton and acts as a scaffold for the cullin3-dependent E3 ubiquitin ligase complex. After poly-ubiquitination, Nrf2 is degraded via the proteasome. There are several ways to induce the nuclear translocation and DNA binding of Nrf2. Of those, the best understood mechanism is the redox-dependent modification of thiol groups in the Keap1 protein, which results in a conformational change locking Nrf2 at Keap1. Newly synthesized Nrf2 can no longer be degraded and translocates to the nucleus. Besides Keap1, caveolin 1, the ubiquitin ligase Skp, cullin, F-box containing complex (SCF), and the retinoid X receptor α (RXRα) also interact with and repress Nrf2. Nrf2 target genes contain so-called antioxidant-responsive elements (ARE) within their promoter regions (reviewed in [2]). The list of Nrf2-regulated genes is further increasing continuously and comprises genes involved in antioxidant defense, NADPH regeneration, glutathione synthesis, and drug detoxification, as well as in metabolic control, including carbohydrate and lipid metabolism [3]. Nrf2 has previously been shown to be modulated by changes in the cellular status of single TEs, e.g., Zn. Zn binding triggers a conformational switch in the cullin3 substrate adaptor function of Keap1, thus Nrf2 becomes stabilized and can activate the transcription of target genes [4]. Furthermore, Zn modulates the activity of several kinases and phosphatases, which accordingly enhances Nrf2 activity (Table 1).

Vice versa, Nrf2 target genes belong to the group of selenoproteins or are involved in regulating the systemic TE status (Table 1). This has already been studied for Fe [3]. In mice, Nrf2 is activated in response to increased hepatic Fe levels [5]. Accordingly, Nrf2 protects the murine liver against the toxicity of dietary Fe overload by preventing cell death of hepatocytes and enhancing Fe release [6]. During inflammation, Nrf2 induces ferroportin (Fpn1), the sole Fe exporter, to enhance Fe efflux from macrophages or enterocytes [7]. Several Fe transport and binding proteins, like Fpn1, hepcidin (Hamp), and ferritin, are Nrf2 target genes [8]. In addition, key enzymes of heme biosynthesis are induced via Nrf2 (Table 1). Altogether, this battery of proteins reduces the pool of free intracellular Fe. A comparable approach is the upregulation of MTs, a family of cysteine-rich proteins that bind Zn and Cu via their thiol groups [9]. Another important mediator of Cu homeostasis is the Cu-transporting ATPase 2 (Atp7b), the Wilson’s ATPase. Being primarily expressed in hepatocytes, Atp7b supports the incorporation of Cu into ceruloplasmin (Cp) and enhances the excretion of Cu from the liver into the bile [10,11].

There is substantial cross-talk between Nrf2 and other transcription factors, including the aryl hydrocarbon receptor (AhR), nuclear factor κ-light-chain-enhancer of activated B cells (NF-κB), tumor suppressor protein p53, and Notch making Nrf2, an important factor in regulating immune defense, differentiation, and tissue regeneration, as well as cell death [22]. Another interesting interaction could take place with the metal regulatory transcription factor 1 (MTF1), which senses Cu and Zn and binds to metal-responsive elements (MREs) in the promoter of target genes. These include the zinc transporter solute carrier family 30 member 1 Slc30A1, encoding for ZnT1, as well as MT1 and 2, but also selenoproteins, like Selenoh, Selenow, and TXNRD2, modulators of the Fe status, like Fpn1 and Hamp [23], and Atp7b [24]. In particular, MT genes often contain AREs next to MREs and are activated via Nrf family members. For MT1, this activation has been shown by Nrf1 as well as by Nrf2, especially in response to the cellular Zn status, albeit to a smaller extent [25]. Thus, MTF1 could be a potential link between Nrf2 signaling and regulation of TE homeostasis.

In most previous studies, the authors focused on high concentrations and pro-oxidant effects of overloading single TEs, e.g., Se. However, we have previously shown that Nrf2 becomes activated under conditions of a suboptimal Se status in the duodenum [17] and liver [26] of mice. NAD(P)H quinone dehydrogenase 1 (NQO1) activity was analyzed as one of the most strongly regulated target genes of Nrf2. Most probably, the Nrf2 activation is an attempt to compensate for the reduced expression of antioxidant selenoproteins. This condition has a much higher physiological relevance than Se supplementation because of the suboptimal nutritional Se supply prevailing in Europe [27]. The U-shaped effect on Nrf2 activity observed for Se could also be true for other TEs; however, this has not been studied systematically so far. 

Based on the results obtained for single TEs, we aimed to study the effect of a whole-body Nrf2 knockout (KO) in mice on the systemic TE status of Fe, Se, Cu, and Zn. A suboptimal Se status results in limited expression of Se-sensitive selenoproteins. As reduced expression of selenoproteins, such as TXNRD1, under Se-deprived conditions further enhances Nrf2 activity [28], we also studied the combination of Nrf2 KO mice with feeding diets that provide either a suboptimal, adequate, or supplemented amount of Se. Focusing only on wild type (WT) mice with different Se statuses allowed the question of whether changes in a single TE (in this case Se) affect the homeostasis of three other TEs to be addressed. In addition to markers for the TE status, TE-related Nrf2 target genes were analyzed in the liver of those mice.

## 2. Materials and Methods

### 2.1. Animal Experiment

Animal experiments were approved by the ethics committee of the Ministry of Agriculture and Environment (State Brandenburg, Germany) and all methods were carried out in accordance to permission number V3-2437-29-2012. Nrf2 KO mice on a C57BL/6J background were kindly provided by Masayuki Yamamoto (Tohoku University Graduate School of Medicine) and genotyped as previously described [29]. Adult male and female mice were used for the animal experiments that were group-housed and random-caged with ad libitum access to a standard chow diet (Ssniff, Soest, Germany, Table 2), with an Se content of 0.3 mg/kg diet, deionized water, 23 °C, and a 12:12 h dark:light cycle. Those mice were sacrificed at an age of 6 months. 

In the second experiment, male WT and Nrf2 KO mice were weaned onto a diet based on torula yeast (Altromin C1045; Lage, Germany, Table 2) with a low basal selenium content of 0.03 mg/kg. For the selenium adequate (+Se) and the supplemented (++Se) diets, the basal chow was enriched with L (+)-selenomethionine (Fisher Scientific, Schwerte, Germany) to a final selenium content of 0.15 or 0.6 mg/kg, respectively. The Se, Fe, Cu, and Zn concentration of the diets was measured by ICP-MS/MS. Diets were fed for 6 weeks until an age of 10 weeks before mice were anesthetized by isoflurane (Abbot, Wiesbaden, Germany), and blood was withdrawn by heart puncture. Plasma was obtained after centrifugation of the blood for 15 min (1200× *g* and 4 °C). 

### 2.2. ICP-MS/MS Analysis of TE

Frozen tissue (liver or duodenum) was pulverized using a TissueLyser (Qiagen, Hilden, Germany) for 2 × 30 s at maximum speed. Feed samples were pulverized by mortar and pestle. About 50 mg of each sample were weighed precisely into polytetrafluoroethylene (PTFE) microwave vessels. For mouse tissue, the variance was found to be far below 5% between replicates of the same mouse (data not shown); therefore, only one replicate was analyzed to preserve tissue for other experiments. However, in the case of obvious outliers, the sample was digested and analyzed again. Due to high in-batch variance in the chow diets from some manufacturers, at least three independent replicates were prepared in the case of feed samples. For tissue samples, 1000 µL of concentrated HNO_3_ (65%, suprapure, Merck, Darmstadt, Germany), 50 µL of a solution containing 100 µg Rh/L (made from 10 mg/L single-element stock solution, Carl Roth, Karlsruhe, Germany) as the internal standard, and 950 µL ultrapure water were added. For feed samples, 900 µL of concentrated HNO_3_, 250 µL of H_2_O_2_ (30%, Merck/Sigma-Aldrich, Darmstadt, Germany), and 810 µL of ultrapure water were added. In addition, 20 µL of a solution containing 1000 µg Rh/L and 20 µL of 10,000 µg ^77^Se/L (made from a 10,000 mg/L stock solution, prepared from isotopically enriched ^77^Se (97.20 ± 0.20% ^77^Se; 0.10% ^74^Se; 0.40 ± 0.10% ^76^Se; 2.40 ± 0.10% ^78^Se; 0.10% ^80^Se; 0.10% ^82^Se as certified by Trace Sciences International, ON, Canada), purchased from Eurisotop SAS (Saarbrücken, Germany), were added as internal standard or isotope dilution standard, respectively. The samples were then digested in a Mars 6 microwave digestion system (CEM, Kamp-Lintfort, Germany) by heating to 200 °C over a period of 10 min and holding this temperature for 20 min. In each digestion, two blank samples and 50 mg of certified reference material ERM-BB 422 (fish muscle) or ERM BB 186 (pig kidney, Merck/Sigma-Aldrich) were carried along to ensure accuracy of results. Samples were repeated if the recovery for any analyzed element deviated by more than 10% from the reference value and/or was outside the error range of the material. After digestion, samples were quantitatively transferred to 15-mL polypropylene tubes combined with two times 475 µL (tissue) or 1 mL (feed) of ultrapure water from vessel rinsing. The samples were kept at 4 °C until one day prior to measurement, when they were further diluted 1 + 4 in 15-mL polypropylene tubes to give a final concentration of 2.93% HNO_3_, as well as either 2.5 µg Rh/L (tissue) or 1 µg Rh/L and 10 µg ^77^Se/L (feed). Mixed-element calibration standards were made to match the concentration of HNO_3_ and the internal standard in the diluted digests from 1000 mg/L single-element stock solutions (Carl Roth). Calibration ranges were Fe: 5–1000 µg/L, Cu: 0.5–100 µg/L, Zn: 2.5–500 µg/L, Se: 0.05–10 µg/L. For Se isotope dilution analysis (IDA), a solution containing 10 µg ^77^Se/L, as well as a 1 + 1 mixture of 10 µg ^77^Se/L and 10 µg naturally distributed Se/L was prepared. Solutions were then analyzed via ICP-MS/MS (8800 ICP-QQQ-MS, Agilent Technologies, Waldbronn, Germany at 1550 W plasma Rf power, equipped with Ni-cones, MicroMist nebulizer at 1.2 L Ar/min and Scott-type spraychamber) monitoring the following mass to charge ratios (Q1→Q2): He-mode: Fe (56→56), Cu (63→63), Zn (66→66), Rh (103→103); O2-mode: Se (77→93), Se (80→96), Rh (103→103). Elements in He-mode were determined via external calibration after internal standard correction using Rh and Se was also determined either via external calibration (tissue) or via isotope dilution analysis (IDA) (feed) as described previously [31]. The instrument was optimized on a daily basis for maximum sensitivity across the relevant mass range (He: Co (59→59), Y (89→89), Tl (205→205); O2: Co (59→59), Y (89→95), Tl (205→205)), an oxide ratio of <1.5% (^156^(CeO)^+^/^140^Ce^+^), and a doubly charged ratio of <2% (^140^Ce^2+^/^140^Ce^+^), as well as a background of <0.1 CPS prior to measurement.

The applied method for the analysis of TEs in murine plasma has been described previously [32]. In brief, 50 µL of murine plasma were diluted 1 + 9 with a dilution mix (5 vol.-% butanol (99%, Alfa Aesar, Karlsruhe, Germany), 0.05 m.-% Na-EDTA (Titriplex^®^ III, pro analysis, Merck), 0.05 vol.-% Triton™ X-100 (Merck Sigma-Aldrich), and 0.25 vol.-% ammonium hydroxide (puriss. p.a. plus, 25% in water, Fluka, Buchs, Germany)), as well as internal standards (final concentrations: 1 µg Rh/L and 30 µg ^77^Se/L). The diluted sample was then subjected to analysis for Fe, Cu, Zn, and Se (IDA) via ICP MS/MS.

### 2.3. RNA Isolation, Reverse Transcription, and Quantitative Real-Time PCR

The mRNA was isolated from frozen and pulverized (TissueLyser; Qiagen) tissues with the Dynabeads mRNA DIRECT Kit (Life Technologies, Fisher Scientific) according to the manufacturer’s protocol. Reverse transcription (RT) was performed with 150 ng mRNA, 0.15 pmol oligo(dT)15 primers, 1× RT buffer, 700 μM dNTPs, 0.1 mg/mL BSA, 30 U RNasin^®^ (Promega, Mannheim, Germany), and 180 U Moloney murine leukemia virus reverse transcriptase (M-MLV RT, Promega) in a total volume of 45 μL. Real-time PCR was performed in a total volume of 25 μL with 1 μL of 1 + 9 diluted cDNA measured in triplicates using a Mx3005P QPCR System (Agilent). SYBR Green I (Molecular Probes, Eugene, OR, USA) served as the fluorescent reporter. The annealing temperature was 60 °C for all PCR reactions and specificity was confirmed by a melting curve analysis. All PCR products were quantified with a standard curve to correct for differences in PCR efficiencies. Primer sequences (Sigma-Aldrich, Steinheim, Germany) are listed in Table 3. A normalization factor was calculated from the two reference genes, *Epcam* and *Rpl13a*, and used for normalization.

### 2.4. ELISA

Ferritin and transferrin were measured in plasma samples using Mouse Ferritin and Transferrin ELISA (ALPCO, Salem, MA, USA) following the manufacturer’s instruction. Therefore, plasma samples were either diluted 1:20 or 1:200,000, respectively.

### 2.5. Western Blot

To obtain protein lysates, frozen liver samples were homogenized in Tris buffer (100 mM Tris, 300 mM KCl, pH 7.6 with 0.1% Triton X-100 (Serva, Heidelberg, Germany)) using a TissueLyser (Qiagen) for 2 × 30 s at maximum speed. Cellular debris was removed by centrifugation (14,000× *g*, 15 min, 4 °C) and protein concentrations were determined by Bradford analysis (Biorad, München, Germany). After SDS polyacrylamide gel electrophoresis, gels were immunoblotted to nitrocellulose and blots were blocked in 5% non-fat dry milk in Tris-buffered saline containing 0.1% Tween 20 at room temperature for 1 h. The following antibodies were used: Rabbit anti-Ferritin-H (151023, Abcam, Cambridge, UK; 1:500), rabbit anti-MT (192385, Abcam; 1:1000), rabbit anti-Ctr1 (129067, Abcam; 1:2000), rabbit anti-MTF-1 antibody (86380, Novus Biologicals, Centennials, US; 1:250), and rabbit anti-β-Actin (8227, Abcam; 1:10,000). Horseradish peroxidase-conjugated goat anti-rabbit IgG (Chemicon, Hofheim, Germany; 1:50,000) served as secondary antibody. Intensities of identified bands were quantified densitometrically with the Luminescent Image Analyzer LAS-3000 system (Fujifilm, Tokyo, Japan). Protein expression was normalized to β-actin expression or Ponceau staining.

### 2.6. Enzyme Activities

Protein lysates were prepared as described in the section ‘Western Blot’. Measurements of NQO1 [17], TXNRD [33], GPX [34], and glutathione S transferase (GST) [35] activities have been described previously. Briefly, NQO1 activity was examined by a menadione-mediated reduction of 3-(4,5-dimethylthiazol-2-yl)-2,5-diphenyltetrazolium bromide (MTT). TXNRD activity was measured by the NADPH-dependent reduction of 5,5′-dithiobis (2-nitrobenzoic acid) (DTNB). GPX activity was determined in an NADPH-consuming glutathione reductase coupled assay. GST activity was conducted using 1-chloro-2,4-dinitrobenzene (CDNB) as substrate in the presence of reduced glutathione. All measurements were performed in triplicates using 96-well plates and a microplate reader (Synergy2, BioTek, Bad Friedrichshall, Germany).

### 2.7. Statistics

Data are shown as mean + SD. Statistical significance was calculated by GraphPad Prism version 5 (San Diego, CA, USA) using two-way analysis of variance (ANOVA) with Bonferroni’s post-test as indicated in the figure legends. A *p*-value below 0.05 was considered statistically significant.

## 3. Results

**Figure 1:**
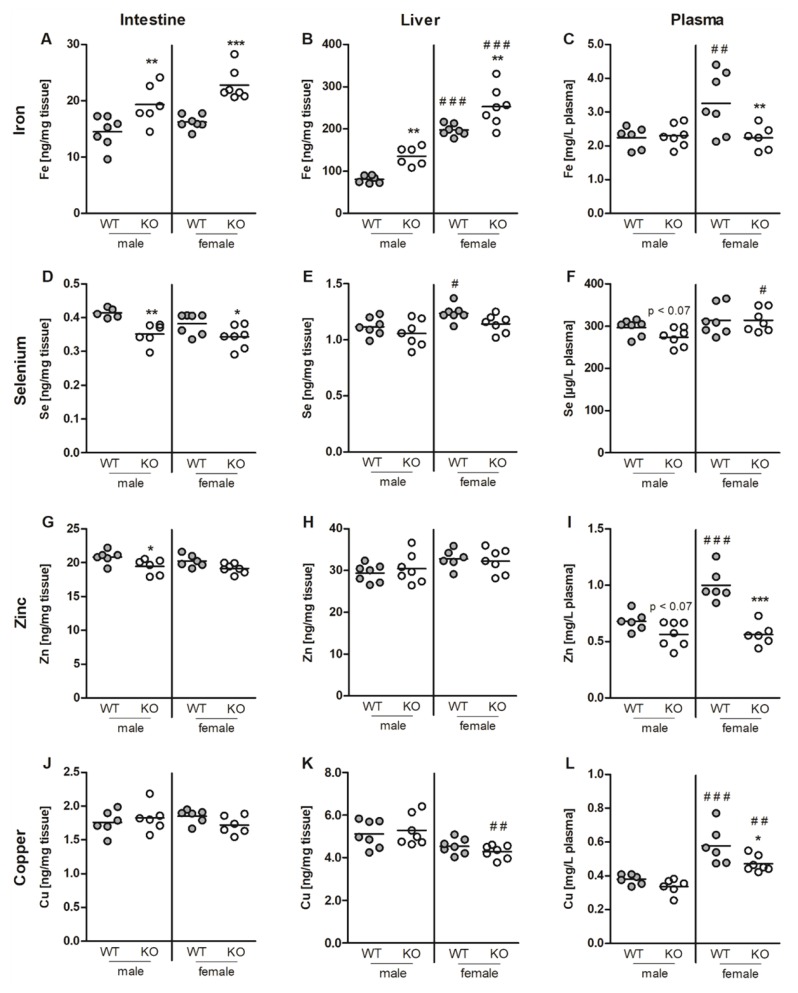
Fe (**A**–**C**), Se (**D**–**F**), Zn (**G**–**I**), and Cu (**J**–**L**) concentrations in the jejunum (**A**,**D**,**G**,**J**), liver (**B**,**E**,**H**,**K**), and plasma (**C**,**F**,**I**,**L**) of six-month-old male and female Nrf2 KO and WT mice fed a standard chow diet with 0.3 ppm Se. The TE profile was analyzed using ICP-MS/MS. Scatter dot plots with mean (*n* = 6–7). * *p* < 0.05; ** *p* < 0.01; *** *p* < 0.001 vs. WT and ^#^ *p* < 0.05; ^##^ *p* < 0.01; ^###^ *p* < 0.001 vs. male (two-way ANOVA with Bonferroni’s post-test).

To address the question of whether Nrf2 not only modulates the status of single TEs, such as Fe, but also of several TEs in parallel, we analyzed Se, Fe, Cu, and Zn in male and female Nrf2 KO mice fed a standard chow diet. TE concentrations were assessed in the intestine, liver, and plasma. Fe was retained more in the liver and small intestine of Nrf2 KO than in WT mice (Figure 1A,B). Consequently, plasma Fe levels were reduced but only in female mice (Figure 1C).

No changes were observed concerning the Fe markers, ferritin and transferrin (Figure S1A,B). In parallel, intestinal Se (Figure 1D) and Zn (Figure 1G) concentrations were reduced in Nrf2 KO compared to WT mice. This was also partially reflected in the Se and Zn plasma and liver values but less consistently. Nrf2-mediated changes of the Cu status appear to be sex specific as only female mice showed lower plasma Cu levels upon loss of Nrf2 (Figure 1L). Overall, Nrf2 reduced the systemic Fe status but increased Se and Zn. Female WT mice had higher plasma levels of Fe, Zn, and Cu. In the liver, amounts of Fe and Se were increased in female mice. In general, chow diets contain high amounts of all TEs, usually at least twice the recommended amounts. Thus, Nrf2-modulated effects might be more pronounced under conditions of limited TE access.

To analyze the role of the Se status on other TEs in combination with loss of Nrf2, both WT and Nrf2 KO mice were weaned onto one of three diets containing suboptimal (0.03 ppm), adequate (0.15 ppm), or supplemented (0.6 ppm) amounts of Se. The experimental set-up was chosen according to previous feeding experiments to efficiently reduce the Se status in the –Se group. For better comparability with previous experiments, only male mice were studied [17]. In addition, the remaining three TEs were reduced in the torula yeast-based diet as compared to the chow diet (Table 2).

**Figure 2:**
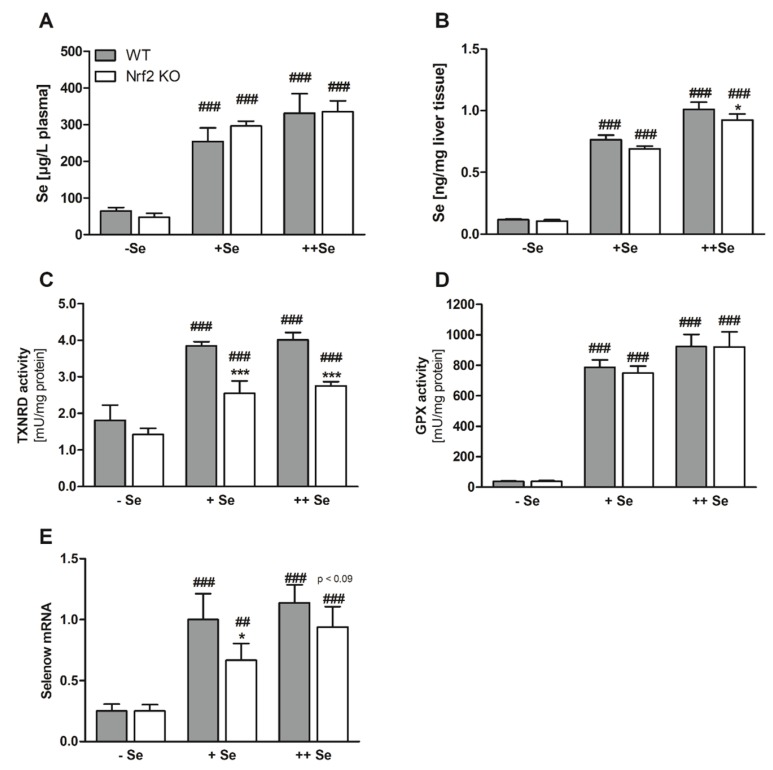
Biomarkers of the Se status. Se concentrations in the plasma (**A**) and liver (**B**) of Nrf2 KO and WT male mice fed diets with defined Se contents (–Se: 0.03 ppm; +Se: 0.15 ppm; ++Se: 0.6 ppm). The TE profile was analyzed using ICP-MS/MS. Enzyme activity of TXNRD (**C**) and GPX (**D**) was analyzed together with mRNA expression of Selenow (**E**) from liver samples of male Nrf2 KO and WT mice. Bars represent means + SD (*n* = 4-5). * *p* < 0.05; *** *p* < 0.001 vs. WT and ^##^ *p* < 0.01; ^###^ *p* < 0.001 vs. –Se (two-way ANOVA with Bonferroni’s post-test).

As expected, the dietary approach successfully modulated the Se status of the different feeding groups (Figure 2A,B). The Se content of the chow diet fed in experiment one was 0.3 ppm and thus between the amount of the +Se (0.15 ppm) and the ++Se (0.6 ppm) diets. Comparing the plasma Se content in both experiments (Figure 1F and Figure 2A) revealed that the +Se diet with 0.15 ppm was already able to set the plasma Se concentration to almost 300 µg/L, which was nearly the same amount as measured in the 0.3 (Figure 1F) or 0.6 ppm Se groups (++Se, Figure 2A).

**Figure 3:**
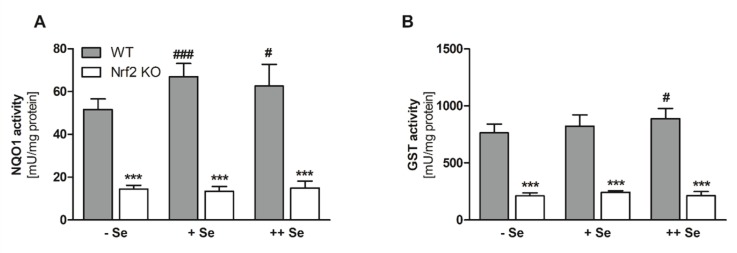
Hepatic enzyme activities as markers for Nrf2 activity. The Nrf2 target gene *NQO1* was measured by an activity assay (**A**). Total enzyme activity of all GST isoforms was measured by an activity assay (**B**). Samples were the liver of Nrf2 KO and WT male mice fed diets with defined Se contents (–Se: 0.03 ppm; +Se: 0.15 ppm; ++Se: 0.6 ppm). Bars represent means + SD (*n* = 4–5). *** *p* < 0.001 vs. WT and ^#^ *p* < 0.05; ^###^ *p* < 0.001 vs. –Se (two-way ANOVA with Bonferroni’s post-test).

In order to confirm that loss of Nrf2 resulted in diminished expression of classical Nrf2 target genes, NQO1 activity (Figure 3A) was analyzed. Enzyme activity was substantially decreased in Nrf2 KO mice. Basal NQO1 mRNA levels were much higher in female than in male mice (Figure S1C). To our surprise, NQO1 activity was not increased under –Se conditions but was significantly decreased in comparison to the +Se or ++Se groups. In Nrf2 KO mice, no Se-dependent effect was detectable. Comparable results were obtained for total GST activity (Figure 3B). 

Besides Nrf2 target genes, selenoprotein expression was also studied. Classical biomarkers of the murine Se status, such as TXNRD and GPX activity, already reached a plateau in the +Se groups and could not be further increased by the ++Se supply (Figure 2C,D). Total TXNRD activity was reduced in Nrf2 KO mice, because TXNRD1 expression is regulated via Nrf2 (Figure 2C). Total hepatic GPX activity, mainly reflecting GPX1 activity, was not affected by the loss of Nrf2 (Figure 2D). Under certain conditions, selenoprotein mRNAs could also serve as biomarkers of the Se status, which is the case for Selenow, showing a four-fold increase in the Se-treated groups in comparison to the –Se group (Figure 2E). Under +Se conditions, Selenow expression was significantly lower in Nrf2 KO than in WT mice and in the ++Se groups there was a trend (*p* < 0.09; Figure 2E). Together with a small reduction of the hepatic Se content, this might indicate that the Se status is lower in Nrf2 KO than in WT mice. 

**Figure 4:**
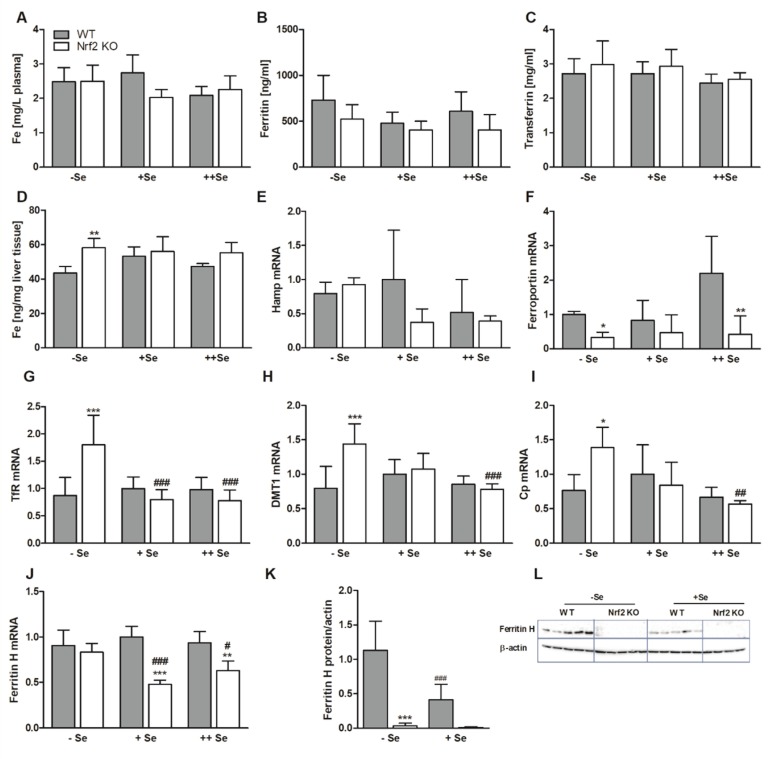
Biomarkers of the Fe status. Fe (**A**), ferritin (**B**), and transferrin (**C**) concentrations in the plasma of male Nrf2 KO and WT mice fed diets with defined Se contents (–Se: 0.03 ppm; +Se: 0.15 ppm; ++Se: 0.6 ppm). Additionally, Fe concentration in the liver (**D**) as well as mRNA and protein expression of Fe-related genes/proteins in the liver were determined by qPCR or western blot, respectively (**E**,**G**–**L**). Ferroportin mRNA was measured in the duodenum (**F**). The TE profile was analyzed using ICP-MS/MS (**A**,**D**). Further Fe plasma parameters were detected by ELISA (**B**,**C**). Bars represent means + SD (*n* = 4–5). * *p* < 0.05; ** *p* < 0.01; *** *p* < 0.001 vs. WT and ^#^ *p* < 0.05; ^##^ *p* < 0.01; ^###^ *p* < 0.001 vs. –Se (two-way ANOVA with Bonferroni’s post-test).

As shown before (Figure 1C and Figure S1A,B), Fe, ferritin, and transferrin plasma levels were unaffected by Nrf2 in male mice (Figure 4A–C). In addition, all three parameters were independent of the Se status. The increased Fe tissue retention described under chow diet conditions (Figure 1) was only significant under –Se conditions in the liver in this case (Figure 4D). To study putative mechanisms for the observed Fe accumulation in the liver, the expression of different Fe-related genes/proteins were tested. 

First, we tested Hamp expression in the liver, because Hamp is the major regulator of Fe homeostasis, which is upregulated in response to an increase in Fe levels. Recently, it has been shown that this upregulation is partially impaired in Nrf2 KO livers [36]. Herein, we could not observe an upregulation in –Se Nrf2 KO livers (Figure 4E). Hamp is known to limit the expression of the Fe exporter Fpn1 in the intestine to reduce systemic Fe levels. Indeed, Fpn1 expression was reduced in the duodenum of both –Se and ++Se Nrf2 KO mice (Figure 4F). Under physiological conditions, Fe is transported in the plasma bound to transferrin, which is taken up by the hepatocytes by binding to the transferrin receptor (TfR). The mRNA expression of TfR was only upregulated in the –Se Nrf2 KO mice (Figure 4G) together with expression levels of the Fe transporter DMT1 (Figure 4H), which is consistent with higher Fe levels in the liver. In the plasma, ferrous Fe is immediately oxidized to ferric Fe by Cu-dependent Cp, and then bound to transferrin. Also, Cp was upregulated in the –Se Nrf2 KO group (Figure 4I). Cp is an acute phase protein, which is known to be sensitive towards inflammation. However, no increase in hepatic inflammatory cells has been detected in Nrf2 KO mice previously [37]. Intracellularly, Fe is efficiently bound to ferritin. The subunit ferritin H (FTH) is regulated by Nrf2 [16]. Herein, mRNA of FTH was only reduced under +Se and ++Se conditions (Figure 4J). However, ferritin H protein levels were almost undetectable also in –Se Nrf2 KO mice (Figure 4K,L). In addition, ferritin H protein was upregulated under –Se conditions in WT mice in comparison to +Se WT mice.

**Figure 5:**
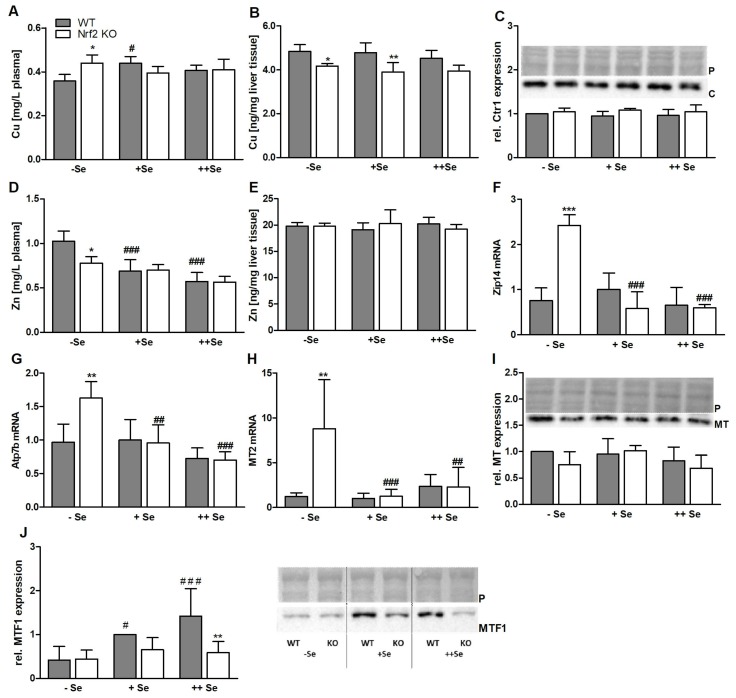
Biomarkers of the Cu and Zn status. Cu and Zn concentrations in the plasma (**A**,**C**) and liver (**B**,**D**) of male Nrf2 KO and WT mice fed diets with defined Se contents (–Se: 0.03 ppm; +Se: 0.15 ppm; ++Se: 0.6 ppm), analyzed by ICP-MS/MS. Additionally, mRNA (**F**–**H**) and protein expression (**C**, **I**–**J**) of Cu- and Zn-related genes/proteins in the liver of these animals were determined. Western blots were normalized to the Ponceau staining (P). Bars represent means + SD (*n* = 4–5). * *p* < 0.05; ** *p* < 0.01; *** *p* < 0.001 vs. WT and ^#^ *p* < 0.05; ^##^ *p* < 0.01; ^###^ *p* < 0.001 vs. –Se (two-way ANOVA with Bonferroni’s post-test). C = Ctr1.

As in the previous experiment (Figure 1), there was no effect of Nrf2 on hepatic or plasma Cu levels under ++Se conditions, but under –Se and +Se conditions hepatic Cu levels were reduced (Figure 5B) while plasma values were increased in the –Se Nrf2 KO group (Figure 5A). The latter obviously resulted from lower Cu levels of –Se WT mice in comparison to +Se WT mice. Higher expression levels of Atp7b (Figure 5G) might be the reason for lower Cu levels in –Se Nrf2 KO mice, while at the same time, higher Cu plasma levels could be explained by more efficient binding of Cu to Cp (Figure 4I) being excreted from hepatocytes. The Cu transporter 1 (Ctr1) is important for Cu as well as Zn absorption in the intestine; however, hepatocytes also express Ctr1 in relevant amounts to take up Cu from the circulation. Ctr1 protein expression was completely unaffected by the Nrf2 genotype or Se supply (Figure 5C). 

As seen before, hepatic Zn levels were neither affected by Nrf2 nor by Se status (Figure 5E). However, plasma concentrations were substantially reduced in Nrf2 KO mice under –Se conditions (Figure 5D). There was a concentration-dependent decrease of plasma Zn values with an increasing Se supply. In contrast to other members of the Zip family, Zip14 transports not only Zn but also Fe. Herein, intracellular Fe concentrations were increased in –Se Nrf2 KO mice, and at the same time, Zip14 mRNA levels were upregulated under these conditions (Figure 5F). Also, MT2 shows a very similar mRNA expression pattern (Figure 5H) to Zip14. It was highly upregulated in –Se Nrf2 KO livers. However, western blots with an antibody against all MT isoforms could not confirm the effect observed for MT2 mRNA expression (Figure 5I). As a potential mechanistic link between regulated genes and the Nrf2 and Se status, MTF1 expression was analyzed in the liver. MTF1 was significantly downregulated in ++Se Nrf2 KO mice (Figure 5J), but MTF1 levels declined with reduction of the Se status and thus the Nrf2 KO effect was lost under +Se and –Se conditions.

## 4. Discussion

It is well established that the transcription factor Nrf2 is an important mediator of Fe homeostasis [8]. In this study, we addressed the question of whether other trace elements, such as Se, Zn, and Cu, are modulated by Nrf2 as well. A reduction of Nrf2 levels and responsiveness is a relevant health condition that physiologically takes place during aging [38]. Thus, the question arises whether age-specific changes in TE profiles [39] might be related to Nrf2. Recently, it has been examined that Nrf2 activity levels strongly differ in the liver of male and female mice [40]. Also, herein, we were able to show that basal NQO1 mRNA expression in WT livers is much higher in female than in male mice (Figure S1C). Thus, sex differences of TEs could be attributed to higher Nrf2 activity as well. All three TEs, Fe, Cu, and Zn, were indeed higher in the plasma of female than in male mice (Table 4), but the underlying mechanisms are unclear so far. Also, MT2 mRNA levels were substantially higher in female livers but at the same time independent of the Nrf2 status (Figure S1E).

We observed an increase in Fe tissue levels upon loss of Nrf2 (Figure 1A,B and Figure 4D). Vice versa, Nrf2 protects the murine liver against dietary Fe overload by enhancing Fe release [6]. Combining a genetic mouse model for hereditary hemochromatosis with an Nrf2 KO results in hepatic fibrosis, which could otherwise be prohibited by upregulation of Nrf2 target genes [41]. Furthermore, under conditions of nutritional steatohepatitis, Nrf2 inhibits hepatic Fe accumulation and thereby counteracts oxidative stress [42]. Recently, it has been shown that Fe-induced Nrf2 activation enhances bone morphogenetic protein 6 (Bmp6) signaling, which upregulates hepcidin expression to fine-tune Fe homeostasis [36]. One of the first observations indicating a change in Fe homeostasis in Nrf2 KO mice was the finding that Nrf2 KO mice have abnormally white teeth in comparison to WT mice due to defective Fe utilization during tooth development [43]. Higher Fe tissue levels can be attributed to the Fe exporter Fpn1, which was downregulated in male Nrf2 KO mice (Figure 4F and Figure S1D), and to the hepatic Fe importers, TfR and DMT1 [44], which were upregulated in –Se Nrf2 KO mice (Figure 4G,H). Also, Zip14 was strongly induced in –Se Nrf2 KO mice (Figure 5F). Zip14 was originally described as a Zn importer with the highest expression in the jejunum and liver, but it is now established that it transports further TEs, such as Fe [45]. Under conditions of Fe depletion, Zip14 membrane localization is impaired based on post-translational modifications [46]. Under physiological conditions, Fe is mainly transported bound to transferrin. Thus, TfR appears to be of major relevance for Fe uptake into the liver. To get an idea of the putative crosstalk between several TEs, we included DMT1 and Zip14, as those not only transport Fe but also additional TEs. Usually, the intracellular free labile Fe pool is tightly regulated. One of the most important regulating proteins is ferritin, which is able to bind up to 4,500 Fe atoms in its core [47]. The amount of intracellular ferritin H was strongly reduced in Nrf2 KO mice (Figure 4J–L), especially on the protein level, indicating that the labile free Fe pool is substantially increased under those conditions. Ferritin can be secreted from both hepatocytes and Kupffer cells to contribute to plasma ferritin levels in addition to the relevant amounts secreted by macrophages [48]. Surprisingly, plasma ferritin levels stayed unaffected by the Nrf2 genotype (Figure 4B and Figure S1A). This might be explained by the fact that plasma ferritin mostly consists of the ferritin L subunit and not H [49], even though ferritin L has been identified as an Nrf2 target gene as well [50]. Based on the observed substantial downregulation of ferritin H, effects of Fe on the liver are supposed to be stronger than detected. Eventually, Fe availability to the systemic circulation is also reduced in Nrf2 KO mice, counteracting the loss of Nrf2-mediated limitation of the intracellular free Fe pool. Indeed, intestinal Fpn1 expression was reduced in Nrf2 KO mice (Figure 4F), indicating that absorbed Fe might be retained there and released back into the intestinal lumen when enterocytes go into apoptosis. In line with this, Fe plasma levels were reduced in Nrf2 KO mice but only in females (Figure 1F). 

In parallel to Fe, intracellular levels of Se and Zn were affected by loss of Nrf2 as well. In this case, both were reduced. Also, plasma Cu levels were slightly reduced in Nrf2 KO mice (overview in Table 4). Overall, these effects were rather small. For Se, the small reduction in liver Se levels could not be confirmed by analyzing selenoproteins, which respond very sensitively towards changes in the Se status. This was the case for total GPX activity. Only Selenow mRNA expression, which might be a useful additional biomarker for the Se status [51], was slightly reduced in Nrf2 KO mice under +Se conditions (Figure 2E). Thus, Nrf2 does not appear to be a major regulator of the Se status.

Comparable to Zip14, mRNA levels of Atp7b, the essential ATPase for Cu export into the bile [10], were upregulated under –Se Nrf2 KO conditions (Figure 5F,G). Additionally, DMT1 (Figure 4F) and MT2 revealed a similar expression pattern (Figure 5H), which could not be confirmed on the protein level when using an antibody capable of detecting all MT isoforms (Figure 5I). MT isoforms are cysteine-rich proteins that efficiently bind Cu and Zn to reduce the amount of both TEs in their free form [9]. Feeding of rats with the Nrf2 activator sulforaphane resulted in a robust induction of genes encoding for MT-1/2 and MT1a [52]. The MT1 promoter contains an ARE that is activated by Nrf1 and Nrf2, but in the latter case, not to the same extent. In Nrf1 KO mice, basal levels of both MT1 and MT2 genes were reduced [25]. Herein, we did not detect any downregulation of MT2 mRNA upon loss of Nrf2. As several genes (*MT2*, *Atp7b*, *Zip14*, *Cp*, *TfR*, and *Dmt1*) showed a comparable expression pattern, being induced specifically under –Se Nrf2 KO conditions, the question arose if there is a common regulator. One possibility would be MTF1, which regulates MT expression in response to Zn or Cu [53]. Recently, it has been shown that the disruption of an MTF1 binding site by a homozygous variant in the promoter of Atp7b likely causes Wilson disease [24]. In addition, the induction of Fpn1 transcription by MTF1 has been shown [54]. However, there was no detectable MTF1 activation in –Se Nrf2 KO livers. In contrast, MTF1 was upregulated in a concentration-dependent manner as a response to Se. Only under ++Se conditions, an Nrf2 genotype effect was detectable, showing lower MTF1 levels upon loss of Nrf2. Thus, MTF1 expression does not provide an obvious explanation for the observed mRNA expression pattern of some MTF1 target genes.

Besides DMT1, Ctr1 is the main universal Cu importer in mammalian cells. A KO of Ctr1 in the intestine resulted in peripheral Cu hypoaccumulation. In parallel, hepatic Fe levels were upregulated [55]. Expression of Ctr1 was unaffected by the Nrf2 genotype and hepatic Se levels (Figure 5C). Furthermore, an Se-dependent reduction of plasma Zn concentration was observed (Figure 5D). An intestine-specific Ctr1 KO did not modulate the systemic Zn status [55], while liver-specific Ctr1 KO mice showed a transient increase in hepatic Zn levels but not in serum [56]. Taken together, homeostasis of Zn and Cu also appears to be regulated rather independently of Nrf2. However, it is possible that there are short-term effects of Nrf2 on TE homeostasis, which might be undetectable in constitutive KO mice because of putative adaptation processes in response to a loss of Nrf2 over time.

We and others have shown previously that a suboptimal selenium supply results in Nrf2 activation predominantly in the liver and intestine of mice [17,57]. This can be attributed mainly to low levels of the selenoproteins TXNRD1 and GPX4, as single KOs of one of these two selenoproteins also activate Nrf2 [28,58,59,60]. Patients with Kaschin–Beck disease, a disease diagnosed under conditions of Se deficiency, have been characterized by higher expression of Nrf2 and its target gene *Hmox1* in whole blood samples as compared to healthy controls, indicating potential Nrf2 activation in humans under Se deficient conditions [61]. However, in this case, we could not confirm previous results and observed no Nrf2 activation indicated by NQO1 and GST activity in the liver (Figure 3) or intestine (Figure S2) of mice fed an –Se diet, even though the levels of TXNRD and GPX activity were in a comparable range to previous experiments [17,26]. Only the Nrf2 target gene *ferritin H* was clearly upregulated in –Se WT livers and was almost lost in Nrf2 KO mice (Figure 4K,L). This phenomenon has also been described in another recent study analyzing the response to lifelong dietary Se interventions in mice. Additionally, in this study, no effect of an Se-deficient diet could be observed on hepatic Nrf2 response genes considering whole transcriptome analyses [62]. Another study found that Se deficiency affected the expression of neither Hmox1 nor NQO1 [63]. In those two studies, and similar to our study, Se deficiency decreased the expression of important selenoproteins but did not activate Nrf2. Thus, it has been suggested that a low Se status interacts with another dietary or environmental component to regulate the Nrf2 response but is not sufficient by itself [62].

Therefore, the initial aim of studying the crosstalk between Nrf2 and selenium status in modulating three other TEs is difficult to address under the present conditions. Herein, it is relatively clear that Se effects observed on Zn appear to be regulated independently of Nrf2. However, when only considering WT mice, we could still draw conclusions towards the role of the Se status on TE status of Zn, Fe, and Cu. Most strikingly, Zn plasma levels were higher in mice with low Se status and vice versa (Figure 5D). In comparison to that, Cu and Fe were rather unaffected by Se. At the same time, hepatic Zn levels were unaffected by the Se status, indicating that Zn appears to be taken up by other tissues besides the liver when Se levels are rising. As MTF1 shows the complete opposite effect than plasma Zn levels and also as most of the MTF1 target genes, it might be an attempt to compensate for the low Se status. Any underlying mechanisms, however, are unclear so far.

## 5. Conclusions

Overall, only Fe was substantially regulated in response to Nrf2 while the impact of Nrf2 on homeostasis of Se, Cu, and Zn appeared to be rather marginal. Nevertheless, crosstalk between Se and MTF1 is a promising idea that needs to be followed up in the future and might provide an explanation for the observed counter regulation of plasma Se and Zn levels.

The mammalian ionome has been evaluated in 26 species and across several tissues [64]. In this study, Zn levels in the liver and kidney were positively correlated with maximum lifespan while hepatic Se was negatively correlated with longevity, albeit in a relatively weak manner. The Nrf2 responsiveness is reduced during aging, which provides a putative explanation for changes in TE profiles in the elderly and for TE effects on longevity. We have recently shown in a reinvited sub-cohort of the EPIC Potsdam study that Cu and Fe serum levels increased over time, while Zn and Se levels showed an age-dependent decline [39]. This is supposed to be associated with a reduction in Nrf2 activity for which, herein, Nrf2 KO mice were used as a model. Also, in Nrf2 KO mice, Se and Zn levels were reduced; however, there was no upregulation but rather a slight downregulation of the systemic Cu status (Table 4). Systemic Fe levels increased in Nrf2 KO mice but that was shown herein for intracellular amounts and not for plasma biomarkers as done in the EPIC study. Thus, it needs to be further clarified how age-dependent changes in the TE status are modulated on the molecular level.

## Figures and Tables

**Table 1:** Effects of single trace elements on Nrf2 signaling (overview in [12]).

TE	Nrf2 Pathway Activity	TE-Related Nrf2 Target Genes
Cu	CuCl_2_ activates Nrf2; Nrf2 is crucial for MRE/ARE-mediated transcription in response to Cu [13]	MT1/2, SOD1
Fe	cytotoxic concentrations of Fe activate Nrf2 in murine primary astrocytes [14] and hepatocytes [6]	Fpn1, heme oxygenase (Hmox1) [15], Hamp, ferritin (FTH-1, FTL) [16], heme transporter (Slc48a1/HRG1), ferrochelatase (Fech), biliverdin reductases (BlvrA/B)
Se	a suboptimal Se status activates Nrf2 in mice [17]; high selenite concentrations enhance Nrf2 target gene expression [18]	GPX2 [19], TXNRD1 [20]
Zn	Zn upregulates Nrf2 function, e.g., via phosphorylation signals [21]	MT1/2, SOD1

**Table 2:** Estimated trace element requirements of mice [30] and trace elements in the diets used.

TE	Requirement (mg/kg)	Chow (Ssniff) (mg/kg)	Altromin C1045 (mg/kg)
Cu	6	8.8	2.7
Fe	35	215	151
Se	0.15	0.3	0.03
Zn	10	97	57

**Table 3:** Primer sequences (5′→3′).

Gene	RefSeq-ID	Sequence
Atp7b, ATPase copper transporting beta	NM_007511.2	CAGATGTCAAAGGCTCCCATTCAG CCAATGACGATCCACACCACC
Cp, ceruloplasmin	NM_001276248.1	GTACTACTCTGGCGTTGACCC TTGTCTACATCTTTCTGTCTCCCA
DMT1, divalent metal transporter 1	NM_001146161.1	CTCAGCCATCGCCATCAATCTC TTCCGCAAGCCATATTTGTCCA
Epcam, epithelial cell adhesion molecule	NM_008532.2	TCATCGCTGTCATTGTGGTGGT TCACCCATCTCCTTTATCTCAGCC
Fpn1, ferroportin	NM_016917.2	CTGGTGGTTCAGAATGTGTCCGT AGCAGACAGTAAGGACCCATCCA
Fth1, ferritin heavy polypeptide 1	NM_010239.2	CGCCAGAACTACCACCAGGA TTCTTCAGAGCCACATCATCTCGG
Hamp, hepcidin	NM_032541.1	AAGCAGGGCAGACATTGCGA TGCAACAGATACCACACTGGGA
MT2, metallothionein 2	NM_008630.2	CTGTGCCTCCGATGGATCCT CTTGTCGGAAGCCTCTTTGCAG
NQO1, NAD(P)H quinone dehydrogenase 1	NM_008706.4	ATGTACGACAACGGTCCTTTCCAG GATGCCACTCTGAATCGGCCA
Rpl13a, ribosomal protein L13a	NM_009438.5	GTTCGGCTGAAGCCTACCAG TTCCGTAACCTCAAGATCTGCT
Selenow, selenoprotein W	NM_009156.2	ATGCCTGGACATTTGTGGCGA GCAGCTTTGATGGCGGTCAC
Tfrc, transferrin receptor	NM_011638.4	GGCTGAAACGGAGGAGACAGA CTGGCTCAGCTGCTTGATGGT
Zip14, solute carrier family 39 member 14	NM_001135151.1	GCCTCACCATCCTGGTATCCGT AGCAGACGAGGCATGAGTCTGG

**Table 4:** Effects of Nrf2 genotype, sex, and a suboptimal Se status on homeostasis of Fe, Zn, and Cu.

TE	Nrf2 Genotype (KO vs. WT)	Sex in WT Mice (Female vs. Male)	Se Effect in WT Mice (–Se vs. +/++Se)
Cu	intracellular Cu → plasma Cu (↓)	plasma Cu ↑	plasma Cu →
Fe	intracellular Fe ↑ plasma Fe biomarkers →	liver and plasma Fe ↑	only hepatic ferritin H ↑
Se	intracellular Se ↓ plasma Se →	liver Se ↑	↓ as expected
Zn	intestinal and plasma Zn ↓ liver Zn →	plasma Zn ↑	plasma Zn ↓

## Supplementary Materials

The following are available online at https://www.mdpi.com/2072-6643/11/9/2112/s1, Figure S1: Ferritin and transferrin concentrations in the plasma and mRNA expression in the livers of male and female Nrf2 KO and WT mice fed a standard chow diet. Figure S2: NQO1 mRNA (A) and activity (B) in the duodenum of male Nrf2 KO and WT mice fed diets with defined Se content (–Se: 0.03 ppm; +Se: 0.15 ppm; ++Se: 0.6 ppm).
