# Supplementary material for: Crosstalk of Nrf2 with the Trace Elements Selenium, Iron, Zinc, and Copper
Source: Nutrients. 2019 Sep 5;11(9):2112. doi: 10.3390/nu11092112 (PMC6770424; doi:10.3390/nu11092112)
Supplement: Supplementary file 1 [file nutrients-11-02112-s001.pdf]

Supplementary

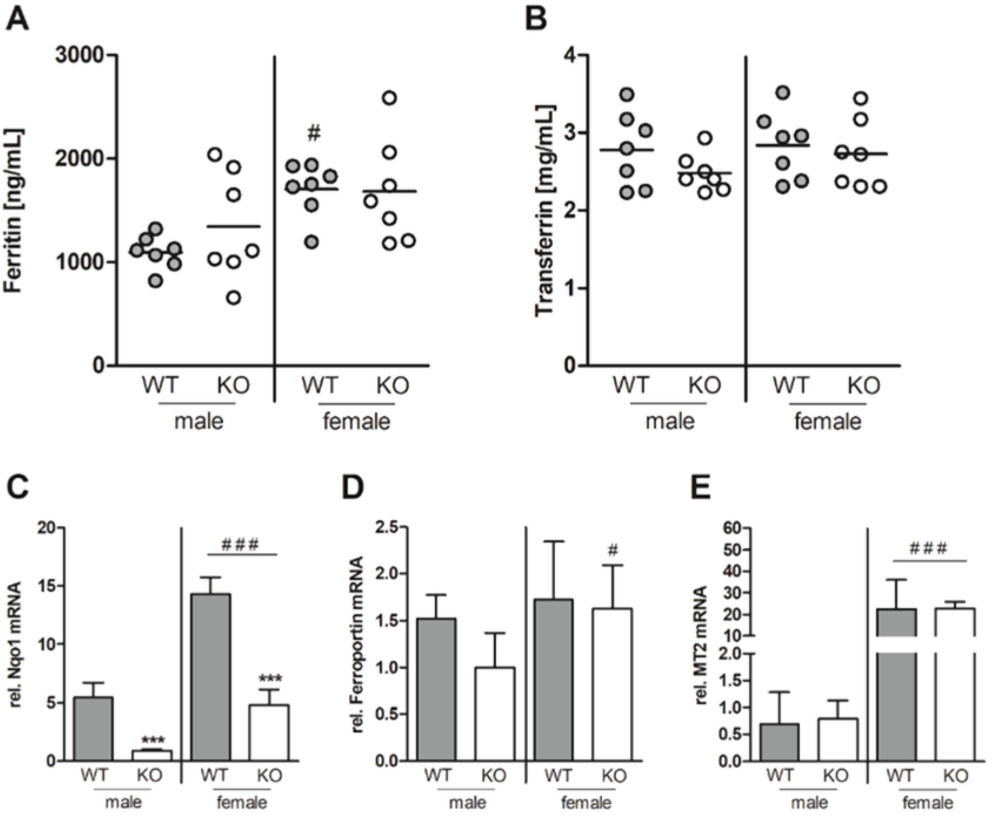

Figure S1. Ferritin and transferrin concentrations in the plasma and mRNA expression in the livers of male and female Nrf2 KO and WT mice fed a standard chow diet. Fe parameters were detected by ELISA (**A**,**B**). mRNA expression of Nqo1, Fpn1, and MT2 was analyzed by qPCR (**C**–**E**). Scatter dot plot with mean (*n* = 7). Bars represent means + SD (*n* = 6–7). *** $p < 0.001$ vs. WT and # $p < 0.05$; ### $p <$ 0.001 vs. male (two-way ANOVA with Bonferroni's post-test).

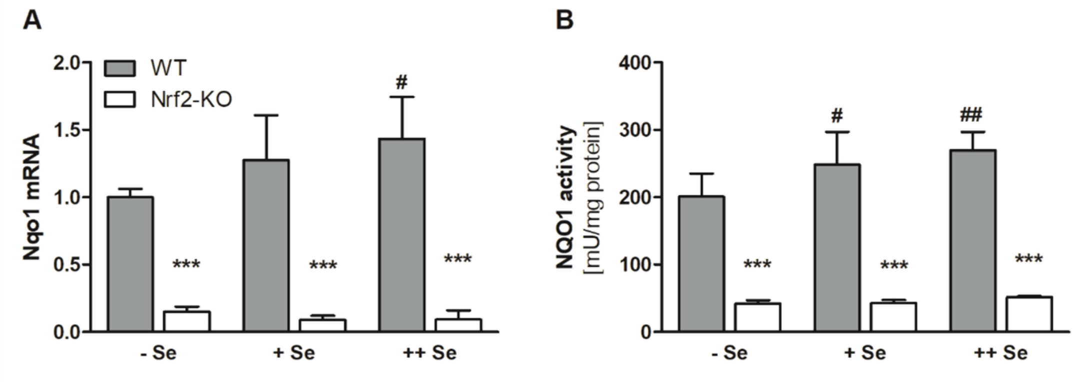

Figure S2. NQO1 mRNA (**A**) and activity (**B**) in the duodenum of male Nrf2 KO and WT mice fed diets with defined Se content (–Se: 0.03 ppm; +Se: 0.15 ppm; ++Se: 0.6 ppm). mRNA expression was analyzed by qPCR and normalized to Rpl13a and Epcam. Bars represent means + SD (*n* = 3–4). *** $p <$ 0.001 vs. WT and # $p < 0.05$; ### $p < 0.001$ vs. –Se (two-way ANOVA with Bonferroni's post-test).
